# Effects of Dietary Supplementation with Whole Lamb Omasum on Gut Health and Metabolism in Shiba Inu Dogs

**DOI:** 10.3390/vetsci13010058

**Published:** 2026-01-07

**Authors:** Aolong Jin, Shuyu Zhou, Shang Cheng, You Yang, Yawang Sun, Zhipeng Sun, Yongju Zhao, Xiaochuan Chen

**Affiliations:** 1 Chongqing Key Laboratory of Herbivore Science, Chongqing Engineering Research Center for Herbivores Resource Protection and Utilization, Chongqing Engineering Research Center of Higher Education for Herbivore, College of Animal Science and Technology, Southwest University, Chongqing 400715, China; jinao8344@email.swu.edu.cn (A.J.); 17302811527@163.com (S.Z.); youygz@163.com (Y.Y.); syaw507@swu.edu.cn (Y.S.); fsunzhipeng@163.com (Z.S.); 2 Animal Husbandry Technology Popularization Master Station of Chongqing, Chongqing 401121, China; chengshang3@126.com

**Keywords:** whole lamb omasum, Shiba Inu dogs, fecal microbiome

## Abstract

The growing pet economy boosts demand for fiber-enriched functional foods to improve canine gut motility and metabolic health. However, low-bioavailability commercial fibers often falter in high-energy diets. Whole lamb omasum—from grass-fed sheep omasum and gastric contents—repurposes a discarded byproduct for waste reduction and sustainable livestock production. This study evaluated the short-term effects of WLO supplementation on gut health and metabolism in healthy adult Shiba Inu dogs. Twelve dogs were randomly assigned to control or WLO groups in a randomized controlled trial. WLO supplementation significantly reduced fecal scores by 8.91% (*p* < 0.05), increased apparent crude fat and fiber digestibility by 3.70% and 11.55% (*p* < 0.05), and elevated serum IgA by 35.79–36.15% and T-AOC by 30.53–35.71% (*p* < 0.05). Serum metabolome revealed 13 between-group and 8 within-subject differences related to lipid and endocrine modulation. Fecal microbiota analysis indicated enrichment of the Bacillota phylum and *Blautia* genus (*p* < 0.05). These findings support WLO as a functional food that enhances gut and metabolic health in small-breed dogs.

## Simple Summary

Amid growing concerns about antimicrobial resistance and the imperative for sustainable livestock production, nutritional strategies incorporating natural functional feed additives are increasingly recognized as an important means of improving overall health in companion animals. This study explores whole lamb omasum (WLO)—the sheep’s omasum with its original gastric contents—as a sustainable and functional pet food source. In a short-term trial with healthy adult Shiba Inu dogs, adding WLO to their diet improved stool consistency, enhanced fat and fiber digestion, and boosted key health markers. Additionally, WLO supplementation induced beneficial shifts in blood metabolites and gut microbiota, notably increasing the abundance of Bacillota and *Blautia*, which are linked to improved gut health. These findings suggest that WLO is a promising functional ingredient for pet food, promoting digestive health, immune function, and metabolism while contributing to sustainability through the use of livestock by-products.

## 1. Introduction

Optimized nutritional strategies incorporating functional ingredients are increasingly acknowledged as an effective approach to improving immune function and overall health in companion animals, while concurrently supporting more sustainable animal production systems [1,2]. In line with this trend, the pet food industry has increasingly emphasized the inclusion of functional ingredients to promote the health of companion animals, particularly through natural supplements [3]. Whole lamb omasum (WLO), consisting of the sheep’s omasum with its original gastric contents intact (unemptied and unflushed, retaining plant-derived material), serves as a fiber-rich raw ingredient for manufacturing dietary supplements in dogs, repurposing a byproduct typically discarded during slaughter to support waste reduction and sustainable livestock production. Rich in plant-based fibers and protein, post-irradiation WLO may enhance gut function primarily through its fiber content, which promotes intestinal motility [4,5,6]. Sheep stomach by-products are a protein-rich resource with significant potential for nutritional and functional applications [7]. However, the incorporation of slaughter by-products into pet foods also introduces microbial contamination risks that may compromise animal and human health through zoonotic transmission [8]. To mitigate these risks, effective sterilization technologies, such as electron beam irradiation, are essential to achieve microbiological safety while preserving nutritional integrity and palatability, thereby enabling the sustainable utilization of these by-products without compromising food safety [9].

Although previous studies have not directly investigated WLO, existing evidence from canine fiber supplementation trials indicates that fiber sources, such as soluble corn fiber, miscanthus grass fiber, lignocellulose, and dried apple, significantly modulate the gut microbiome composition in dogs [10,11,12,13,14]. The canine gut microbiota constitutes a critical functional ecosystem that plays an essential role in digestion, immune regulation, and metabolic homeostasis, thereby exerting profound effects on overall health [15,16,17,18,19]. Traditional commercial lamb green tripe products are typically processed to remove gastric contents and thus contain minimal fiber. The WLO used in this study, however, was prepared as slices comprising the entire sheep omasum and its gastric contents. By retaining this plant-derived material, the WLO exhibited a higher crude fiber content, offering the potential to modulate gut health and microbiota in dogs. Compared to large-breed dogs, small-breed dogs exhibit distinct gastrointestinal physiological characteristics, resulting in differences in digestion and metabolism [20,21,22,23]. Shiba Inu dogs, as a typical small-breed representative, exhibit a marked sensitivity in their gut microbiota, which may facilitate the observation of microbial compositional changes under certain conditions, such as dietary interventions [24,25]. We hypothesized that WLO supplementation would induce beneficial shifts in gut microbiota composition via its plant-derived fiber content, thereby enhancing serum markers. The primary objective of this study was to evaluate the effects (transient, most likely) of short-term supplementation of WLO on gut health and metabolism in adult Shiba Inu dogs over a 15-day period using between-group and within-subject analyses.

## 2. Materials and Methods

### 2.1. Animals and Ethical Statement

All experimental procedures were designed and conducted in strict accordance with the National Guidelines for the Care and Use of Laboratory Animals in China and were approved by the Southwest University Laboratory Animal Ethics Review Committee (approval No. IACUC-20251124-08). Twelve purebred Shiba Inu dogs (mean age: 2.74 ± 0.21 years; mean body weight: 11.06 ± 0.52 kg; BCS: 4–5/9) were recruited from local breeders and verified owners. All dogs underwent comprehensive physical examinations confirming good health status, with no clinical signs of disease, and were vaccinated and dewormed prior to study initiation. Daily socialization with conspecifics and caretakers was provided.

### 2.2. Diets and Experimental Design

The basal diet used in this experiment was a standard commercial dog food (Rosyfresh, Nanping, China). The ingredient composition and nutrient levels of the basal diet are presented in Table S1. The basal diet met the Association of American Feed Control Officials (AAFCO 2023; 2023 Official Publication. Association of American Feed Control Officials: Champaign, IL, USA, 2023) maintenance nutrient profiles for adult dogs. In accordance with National Research Council (NRC 2006; Nutrient Requirements of Dogs and Cats. National Academies Press: Washington, DC, USA, 2006) recommendations, each Shiba Inu dog received 200 g of feed per day, divided into two meals.

**Figure 1:**
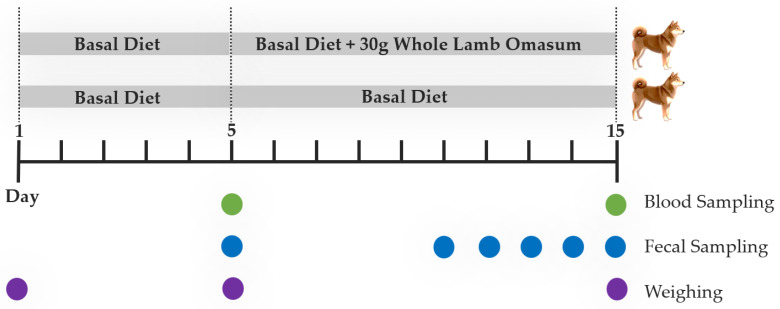
The time flow chart for treatment and data collection.

Twelve healthy adult Shiba Inu dogs were randomly allocated to two groups (*n* = 6 per group). There were no significant between-group differences in baseline age or body weight. Both the control and treatment groups were balanced for sex. The experiment lasted 15 days, comprising a 5-day pre-feeding acclimation period (days 1–5) followed by a 10-day formal feeding phase (days 6–15). During the 5-day acclimation period, all dogs received the basal diet. Beginning on day 6, the control group continued on the basal diet, whereas the treatment group received the basal diet supplemented with 30 g/day WLO, a level selected to comprise no more than 10% of estimated daily caloric requirements, consistent with World Small Animal Veterinary Association (WSAVA) recommendations for treats or supplemental foods in dogs. The WLO was provided first at each feeding to guarantee its complete intake, followed by the basal diet once the WLO allocation was entirely consumed. The WLO was provided in individual 15 g vacuum-packed portions, enabling even distribution of the daily 30 g supplementation (15 g per meal). The experimental design is summarized in Figure 1, and the nutrient composition of WLO is provided in Table 1. For analyses, time-stratified subgroups were defined relative to the onset of formal feeding: CON_Pre (control, days 1–5), CON_Post (control, days 6–15), WLO_Pre (treatment, days 1–5), and WLO_Post (treatment, days 6–15). Samples for microbiota and metabolite analyses were collected at the end of each period to evaluate temporal changes.

### 2.3. Source, Processing, and Safety Assessment of WLO

**Figure 2:**
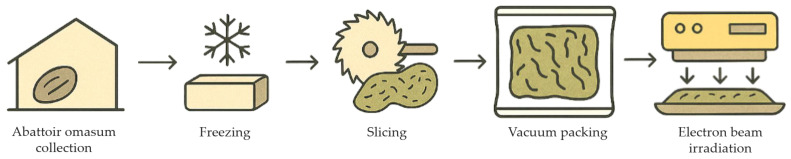
The processing flow of WLO.

The WLO was obtained from 6-month-old Sunite sheep born in March, raised under natural grazing conditions in Xilingol League, Inner Mongolia Zizhiqu, China, and slaughtered in September to ensure a consistent grass-fed condition. The WLO used for animal feeding in this study, all originating from these sheep slaughtered in Xilingol League, were sourced solely through Chongqing Daxibei Beef and Sheep Company (Chongqing, China), where they underwent a standardized processing workflow consisting of five sequential steps: abattoir omasum collection with retention of intact gastric contents (unemptied), freezing, slicing, vacuum packing, and electron beam irradiation at 3.0 kGy (Figure 2). A representative image of the processed WLO is shown in Figure S1. Validation by the China National Accreditation Service for Conformity Assessment verified microbiological safety, including absence of brucellosis and pseudorabies virus. Analyses of the procured WLO adhered to Chinese national standards (GB/SN/T), covering eight indicators: *Salmonella* (GB/T 13091-2018; Determination of *Salmonella* in feeds. National Standardization Administration of the People’s Republic of China: Beijing, China, 2018), *Shigella* (GB/T 8381.2-2005; Determination of *Shigella* in feeds. National Standardization Administration of the People’s Republic of China: Beijing, China, 2005), *Staphylococcus aureus* (GB 4789.10-2016; National food safety standard—Food microbiological examination: *Staphylococcus aureus*. National Health and Family Planning Commission of the People’s Republic of China: Beijing, China, 2016), *Escherichia coli* (GB 4789.38-2012; National Food Safety Standard—Food Microbiological Examination: Enumeration of *Escherichia coli*. Ministry of Health of the People’s Republic of China: Beijing, China, 2012), aerobic plate count (GB 4789.2-2022; National food safety standard—Microbiological examination of food: aerobic plate count. National Health Commission of the People’s Republic of China: Beijing, China, 2022), molds count (GB/T 13092-2006; Enumeration of molds count in feeds. National Standardization Administration of the People’s Republic of China: Beijing, China, 2006), Brucellosis (SN/T 4463-2016; Test of Brucellosis with real-time PCR at frontier port. General Administration of Quality Supervision, Inspection and Quarantine of the People’s Republic of China: Beijing, China, 2016), and Pseudorabies virus (GB/T 35911-2018; Real-time PCR method for detection of pseudorabies virus. National Standardization Administration of the People’s Republic of China: Beijing, China, 2018). All parameters complied with safety limits (Table S2), confirming suitability for feeding.

### 2.4. Food Intake and Body Weight Measurements

Feed intake was continuously recorded for each dog throughout the experimental period. The actual intake was calculated as the amount of feed offered minus the amount of feed remaining. Body weight was measured for all Shiba Inu dogs on days 5 and 15, with measurements performed at consistent times across assessment points.

### 2.5. Fecal Analysis and Digestibility Measurements

Fecal consistency was assessed daily during the final 5 days of the experiment using the Waltham fecal scoring system, which ranges from 1 (hard and dry) to 5 (watery stool) [26]. During the final 3 days of the experiment, fresh fecal pH was immediately measured after preparing a 10% fecal suspension in ultrapure water using a pH meter (EcoTestr pH2, Thermo Fisher Scientific, Waltham, MA, USA). Digestibility was evaluated using the total fecal collection method [27]. Total fecal output was collected daily from all experimental Shiba Inu dogs during the final four days of the 15-day feeding trial. Conventional nutrient components—including crude protein (Kjeldahl method), crude fat (Soxhlet extraction), crude fiber (acid–alkali hydrolysis), ash (muffle furnace at 550 °C), and moisture (oven drying at 105 °C)—were analyzed in triplicate following the AOAC International (AOAC INTERNATIONAL; Official Methods of Analysis of AOAC INTERNATIONAL. Oxford University Press: Oxford, United Kingdom, 2023) standard procedures. Apparent digestibility coefficients for individual nutrients were calculated using the standard equation: Apparent digestibility (%) = [(nutrient intake − fecal output)/nutrient intake] × 100.

### 2.6. Blood Sample Collection and Analysis

On days 5 and 15, following an overnight fast, 5 mL of blood was collected from each Shiba Inu dog via forelimb venipuncture. Samples were allowed to clot for 30 min at room temperature, then centrifuged at 3000× *g* for 15 min at 4 °C to isolate serum, which was aliquoted and stored at −80 °C until analysis. Serum biochemical parameters, including total protein (TP), albumin (ALB), globulin (GLB), total bilirubin (TBIL), aspartate aminotransferase (AST), alanine aminotransferase (ALT), amylase (AMY), creatine kinase (CK), creatinine (CREA), blood urea nitrogen (BUN), glucose (GLU), triglycerides (TG), calcium (Ca), and phosphorus (PHOS), as well as derived ratios (ALB/GLB, BUN/CRE, Ca × PHOS, and AST/ALT), were measured using an automated biochemistry analyzer with commercial reagent kits (SMT-120VP, Seamaty Technology, Chengdu, China). Serum levels of total antioxidant capacity (T-AOC), superoxide dismutase (SOD), and malondialdehyde (MDA) were measured using commercial ELISA kits (BC1315, BC5165, and BC6415, respectively; Beijing Solarbio Science & Technology Co., Ltd., Beijing, China). Immunoglobulin A (IgA) concentrations were quantified using an ELISA kit (ED7O001M; Shanghai Weiao Biotechnology Co., Ltd., Shanghai, China).

### 2.7. Serum Metabolome Analysis

Serum metabolite profiling was performed using an untargeted LC–MS/MS approach following standard sample preparation procedures involving protein precipitation, metabolite extraction, and reconstitution in an organic solvent mixture [28,29]. Analyses were conducted on a UHPLC system coupled to a Q Exactive Hybrid Quadrupole-Orbitrap Mass Spectrometer (Thermo Fisher Scientific, USA) equipped with an ACQUITY HSS T3 column (Waters, Milford, MA, USA) under optimized chromatographic and ESI–MS/MS conditions in negative-ion mode. Compound Discoverer 3.4 software (Thermo Fisher) was used to query the mzCloud and mzVault libraries for peak extraction, alignment, and metabolite identification. For multivariate analysis, MetaboAnalyst 6.0 (https://www.metaboanalyst.ca, accessed on 11 November 2025) was used. In this study, principal component analysis (PCA) and orthogonal partial least squares discriminant analysis (OPLS-DA) were conducted using MetaboAnalyst 6.0. Additionally, volcano plots were generated in MetaboAnalyst 6.0 to visualize differential abundance, incorporating fold-change thresholds and statistical significance for feature prioritization.

### 2.8. Fecal Microbiota Analysis

#### 2.8.1. Fecal Samples

Fecal samples for microbiome analysis were collected on days 5 and 15 of the experiment. Immediately after collection, the samples were placed on dry ice and transported to the laboratory, where they were stored at −80 °C until high-throughput DNA sequencing.

#### 2.8.2. DNA Extraction, Amplification, and Sequencing

Total genomic DNA was extracted from fecal samples using the E.Z.N.A.^®^ Fecal DNA Kit (Omega Bio-tek, Norcross, GA, USA) following the manufacturer’s instructions. DNA quality and quantity were assessed by 1% agarose gel electrophoresis and NanoDrop One spectrophotometry (Thermo Fisher Scientific), and extracts were stored at −80 °C until use. The V3–V4 hypervariable region of the bacterial 16S rRNA gene was amplified with primer pair 338F (5′-ACTCCTACGGGAGGCAGCAG-3′) and 806R (5′-GGACTACHVGGGTWTCTAAT-3′); PCR reactions were performed using a high-fidelity polymerase (FastPfu) with standard buffer, dNTPs and BSA, and cycling conditions of initial denaturation, 27 amplification cycles (denaturation, annealing at 55 °C, extension) and a final extension. PCR products were size-verified on 2% agarose gels, purified using the AxyPrep DNA Gel Extraction Kit (Axygen, Union City, CA, USA), and quantified with a Qubit 3.0 fluorometer (Invitrogen, Carlsbad, CA, USA). Equimolar pooled libraries were sequenced using paired-end 2 × 300 bp reads on an Illumina NovaSeq 6000 platform at Majorbio Bio-Pharm Technology Co., Ltd. (Shanghai, China). Quality control of the raw paired-end sequencing reads was performed with fastp (https://github.com/OpenGene/fastp, accessed on 11 November 2025, version 0.19.6), followed by merging of paired-end reads using FLASH (https://ccb.jhu.edu/software/FLASH/index.shtml, accessed on 11 November 2025 version 1.2.11). Denoising of the sequencing reads was then conducted via the DADA2 plugin in the QIIME2 pipeline, encompassing noise filtering and sequence error correction, removal of chimeras and singletons, and sequence dereplication to generate high-resolution amplicon sequence variants (ASVs) for downstream analyses; the number of sequences retained post-denoising is summarized in Table S3. Further sequence processing and microbial community analyses were carried out on the Majorbio Cloud platform (https://cloud.majorbio.com, accessed on 11 November 2025).

### 2.9. Statistical Analysis

All data were analyzed using GraphPad Prism 9.5 (GraphPad Software, San Diego, CA, USA). Normality and homogeneity of variance were assessed using the Shapiro–Wilk and Levene’s tests, respectively. Between-group differences were evaluated with unpaired t-tests or Mann–Whitney U tests (when assumptions were violated), while within-group differences were analyzed using paired t-tests. Alpha diversity indices and relative bacterial abundances were compared using Welch’s t-test or Wilcoxon rank-sum test as appropriate. Data are expressed as mean ± standard error of the mean (SEM). Differences were considered statistically significant at *p* < 0.05 and highly significant at *p* < 0.01.

## 3. Results

### 3.1. Pre-Feeding Period Body Condition Evaluation

During the 5-day acclimation period before the formal feeding phase, both groups of dogs were fed the same basal diet to establish baseline consistency between the control (CON_Pre) and treatment (WLO_Pre) groups. No significant differences were observed between groups in daily food intake (DM), GE intake, or body weight (Table S4). Baseline values of all blood biochemical, renal, hepatic, metabolic, mineral, and immune/antioxidant parameters showed no significant differences between the CON_Post and WLO_Post groups (Table S5). Additionally, alpha diversity indices (ace, chao, Shannon, Simpson, pielou_e) of the fecal microbiota and beta diversity PCoA analysis showed no significant differences between the CON_Pre and WLO_Pre groups (Table S6 and Figure S2).

### 3.2. Post-Feeding Between-Group Study Results

#### 3.2.1. Food Intake and Body Weight

After 10 days of WLO feeding, no significant differences in daily food intake (DM), GE intake, or body weight were observed between the CON_Post and WLO_Post groups (Table 2).

#### 3.2.2. Fecal Characteristics

The fecal characteristics are presented in Table 3. Compared with the basal diet group (CON_Post), the fecal score was significantly lower in the WLO_Post group (*p* < 0.05). Additionally, supplementation of WLO in the basal diet had no significant effects on fecal pH or fecal dry matter.

#### 3.2.3. Apparent Nutrient Digestibility

The results of the apparent nutrient digestibility are presented in Table 4. No significant differences were observed in the apparent digestibility of dry matter and crude protein among the groups. The dogs fed the basal diet with WLO supplementation (WLO_Post) showed significantly higher crude fat digestibility (from 81.61% to 84.63%) compared with those fed the basal diet without supplementation (CON_Post). The apparent digestibility of crude fiber exhibited highly significant differences between the CON_Post and WLO_Post groups (*p* < 0.05), with a marked increase in the treatment group (from 68.20% to 76.02%). Additionally, the apparent digestibility of calcium and phosphorus also showed no significant differences between the CON_Post and WLO_Post groups.

#### 3.2.4. Biochemistry Parameters

Blood biochemical parameters are reliable indicators of systemic health, metabolic function, and immune status in dogs. In this between-group study, WLO supplementation did not produce detectable effects on plasma proteins (total protein, albumin, globulin, albumin/globulin ratio), liver and kidney enzymes (aspartate aminotransferase, alanine aminotransferase, AST/ALT ratio, amylase, creatine kinase, total bilirubin), renal markers (creatinine), energy metabolites (glucose, triglycerides), minerals (calcium, phosphorus, calcium × phosphorus product), or antioxidant markers (superoxide dismutase, malondialdehyde) compared to CON_Post (Table 5). However, it is noteworthy that several indices showed significant increases in the WLO_Post group (Table 5): blood urea nitrogen increased by 42.01% (from 6.07 mmol/L to 8.62 mmol/L, with a healthy range in dogs of 2.50–9.60 mmol/L; *p* < 0.05), the BUN/creatinine ratio increased by 40.30% (*p* < 0.05), immunoglobulin A increased by 36.15% (*p* < 0.05), and total antioxidant capacity increased by 30.53% (*p* < 0.05). These changes suggest potential improvements in nitrogen metabolism, immune response, and antioxidant defense due to WLO supplementation.

#### 3.2.5. Serum Metabolomic Profile

**Figure 3:**
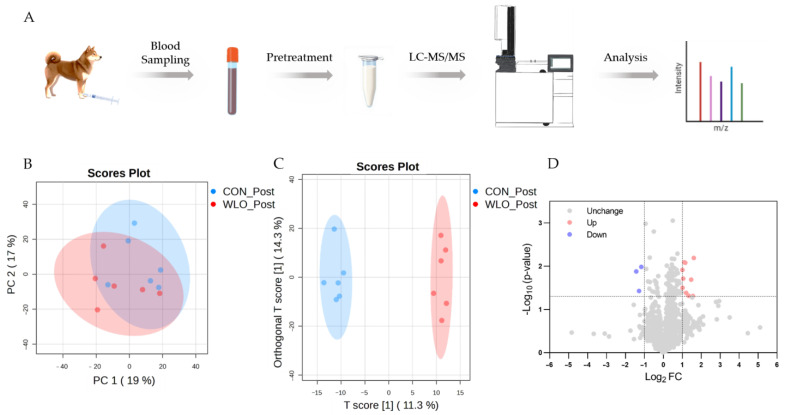
Metabolomics analysis of serum in negative ion mode between the CON_Post and WLO_Post groups. (**A**) Schematic illustrating the metabolomics experimental protocol. (**B**) PCA scatter plot showing the differences between groups based on metabolomics data (*n* = 6). (**C**) OPLS-DA score plot showing the differences between groups based on metabolomics data (*n* = 6). (**D**) Volcano plot of differential metabolites between groups (|log_2_(FC)| > 1, *p*-value < 0.05, *n* = 6).

Metabolomics provided a hypothesis-generating framework for the unbiased detection and quantification of diverse serum metabolites, thereby revealing potential biomarkers and alterations in metabolic pathways in response to the nutritional intervention. In this between-group study, principal component analysis (PCA) and orthogonal partial least squares discriminant analysis (OPLS-DA) were performed as multivariate exploratory analyses to evaluate separation between the CON_Post and WLO_Post groups. PCA showed that serum metabolites from the CON_Post and WLO_Post groups exhibited partial separation along the first principal component, with some overlap indicating residual similarities in metabolic profiles (Figure 3B). OPLS-DA was then applied to examine the clustering of the two groups. The score plot clearly shows that the CON_Post and WLO_Post samples could be successfully distinguished (Figure 3C). To identify metabolites altered by WLO supplementation, volcano plots were generated for serum samples from WLO_Post vs. CON_Post dogs. A total of 1208 metabolites were identified from metabolomics platforms. The criteria used were |log_2_(FC)| > 1 and *p*-value < 0.05. The volcano plot revealed a total of 13 significantly differential metabolites, of which 10 were upregulated and 3 were downregulated (Figure 3D). Detailed metabolite information is presented in Table S7. The significantly altered serum metabolites include phytoestrogens, phospholipids, fatty acid-derived oxylipins, and several plant or xenobiotic-derived compounds—indicating potential perturbations in endocrine regulation and lipid metabolic processes.

#### 3.2.6. Fecal Microbiota

**Figure 4:**
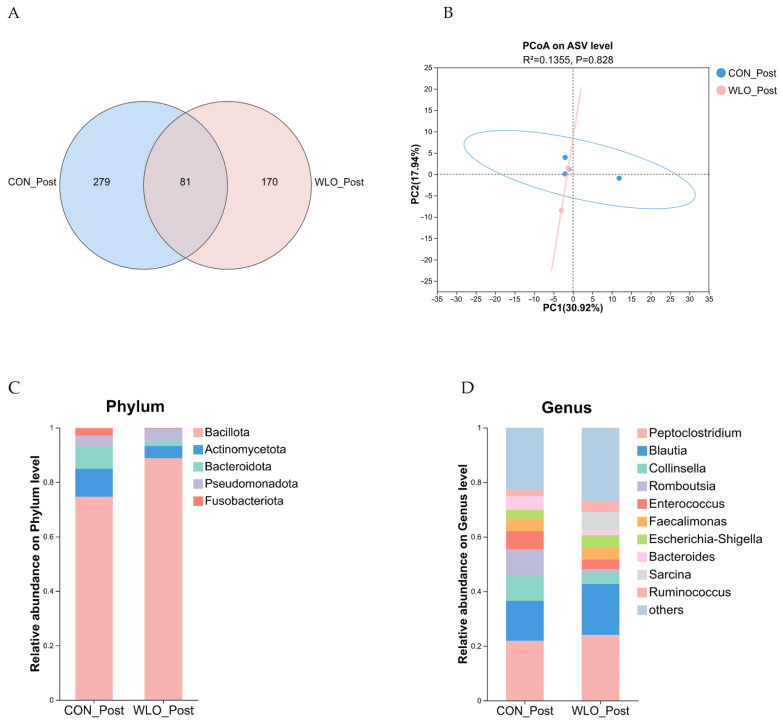
16S rRNA gene sequencing of fecal samples from the CON_Post and WLO_Post groups (*n* = 4). (**A**) Venn diagram illustrating the differentially abundant ASVs. (**B**) Principal coordinates analysis (PCoA) of beta diversity. (**C**) Taxonomic composition of fecal microbiota at the phylum level. (**D**) Taxonomic composition of fecal microbiota at the genus level.

Venn diagrams were used to quantify the shared and unique amplicon sequence variants (ASVs) across multiple samples, providing an intuitive visualization of the compositional similarities and overlaps in ASV profiles from the environmental samples. At the ASV level, species richness in fecal samples from the two groups of Shiba Inu was analyzed to determine the number of shared and unique species between the groups. Overall, the CON_Post group yielded 360 ASVs, while the WLO_Post group yielded 251 ASVs, with 81 ASVs shared between both groups (Figure 4A).

In this between-group study, alpha and beta diversity analyses were applied to comprehensively evaluate the impact of WLO supplementation on the fecal microbiota of Shiba Inu dogs. Alpha diversity indices—including Ace and Chao for estimating community richness, Shannon and Simpson for assessing community diversity, and Pielou’s evenness index for evaluating distribution uniformity—showed no significant differences between groups (Table 6), suggesting that microbial richness, diversity, and evenness remained largely unchanged following WLO supplementation. Consistent with these findings, beta diversity analysis based on Binary Euclidean distance-derived PCoA at the ASV level, coupled with permutational multivariate analysis of variance (ADONIS), demonstrated no significant compositional differentiation between the CON_Post and WLO_Post groups (Figure 4B; R^2^ = 0.1355, *p* = 0.828), as evidenced by substantial overlap in the ordination space. Collectively, these results indicate that WLO supplementation did not exert a detectable influence on the structural stability or compositional architecture of the fecal microbial communities between the CON_Post and WLO_Post groups.

16S rRNA gene sequencing of fecal samples revealed phylum-level alterations after 10 days of WLO supplementation. The five most abundant phyla collectively accounted for over 99% of the microbiota, including Bacillota, Actinomycetota, Bacteroidota, Pseudomonadota, and Fusobacteriota (Figure 4C). In the WLO_Post group, the relative abundance of Bacillota significantly increased by 18.92% compared to the CON_Post group (Table 7; *p* < 0.05), whereas Actinomycetota decreased by 56.36%, Bacteroidota by 78.36%, and Fusobacteriota by 91.54%. Pseudomonadota exhibited a non-significant increase of 16.71%. To investigate microbiota alterations at the genus level, taxonomic annotation was performed at this resolution. The relative abundances at the genus level revealed that the top 8 abundant genera were *Peptoclostridium*, *Blautia*, *Collinsella*, *Romboutsia*, *Enterococcus*, *Faecalimonas*, *Escherichia-Shigella*, and *Bacteroides* (Figure 4D). In the WLO_Post group, the relative abundances of several genera exhibited non-significant changes compared to the CON_Post group (Table 8): *Peptoclostridium* increased by 9.77%, *Blautia* by 27.35%, *Escherichia-Shigella* by 41.67%, whereas *Collinsella* decreased by 55.45%, *Romboutsia* by 87.59%, *Enterococcus* by 45.95%, *Faecalimonas* by 5.70%, and *Bacteroides* by 65.82%.

#### 3.2.7. Spearman Correlation Analysis Between Fecal Bacteria and Differential Metabolites

**Figure 5:**
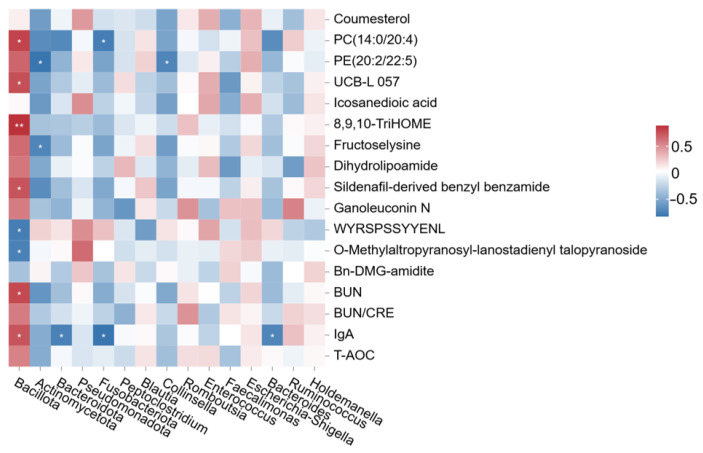
Spearman correlation heatmap between fecal bacteria and differential metabolites. The symbol (*) indicates a significant correlation between fecal bacteria and differential metabolites (* *p* < 0.05, ** *p* < 0.01). Red indicates a positive correlation, and blue indicates a negative correlation.

The correlation between fecal microbiota (at phylum and genus levels) and differential serum metabolites (including biochemical parameters) is shown in the heatmap (Figure 5). As shown, Bacillota exhibited significant positive correlations with PC(14:0/20:4), UCB-L 057, 8,9,10-TriHOME, sildenafil-derived benzyl benzamide, BUN, and IgA, and negative correlations with WYRSPSSYYENL and O-methylaltropyranosyl-lanostadienyl talopyranoside. Actinomycetota exhibited negative correlations with PE(20:2/22:5) and fructoselysine. Bacteroidota exhibited negative associations with IgA, while Fusobacteriota exhibited negative associations with IgA and PC(14:0/20:4). At the genus level, *Collinsella* exhibited negative correlations with PE(20:2/22:5), and *Bacteroides* with IgA. These patterns suggest potential microbiota-driven modulation of lipid, oxidative, and immune-related pathways.

### 3.3. Post-Feeding Within-Subject Study Results

#### 3.3.1. Food Intake and Body Weight

After 10 days of WLO feeding, no significant differences in daily food intake (DM), GE intake, or body weight were observed between the WLO_Pre and WLO_Post groups (Table 9).

#### 3.3.2. Biochemistry Parameters

In this within-subject study, WLO supplementation did not significantly affect plasma proteins (TP, ALB, GLB, ALB/GLB), hepatic and renal enzymes (AST, ALT, AST/ALT, AMY, CK, total bilirubin), renal markers (CREA), energy metabolites (GLU, TG), minerals (Ca, PHOS, Ca × PHOS), or antioxidant indices (SOD, MDA) compared with the WLO_Pre group (Table 10). Notably, several indices showed significant increases in the WLO_Post group (Table 10): BUN increased by 40.16% (from 6.15 mmol/L to 8.62 mmol/L, with a healthy range in dogs of 2.50–9.60 mmol/L; *p* < 0.05), the BUN/CREA increased by 40.75% (*p* < 0.05), IgA increased by 35.79% (*p* < 0.05), and T-AOC increased by 35.71% (*p* < 0.05).

#### 3.3.3. Serum Metabolomic Profile

**Figure 6:**
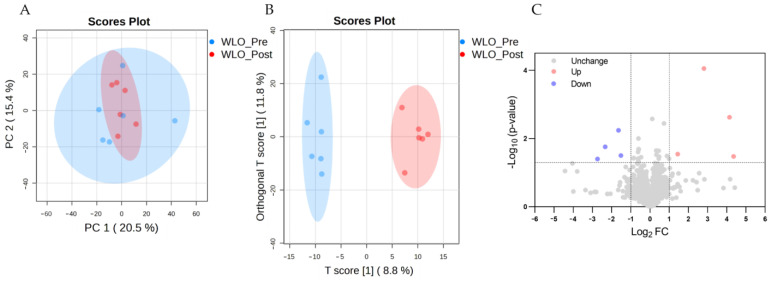
Metabolomics analysis of serum in negative ion mode between the WLO_Pre and WLO_Post groups. (**A**) PCA scatter plot showing the differences between groups based on metabolomics data (*n* = 6). (**B**) OPLS-DA score plot showing the differences between groups based on metabolomics data (*n* = 6). (**C**) Volcano plot of differential metabolites between groups (|log_2_(FC)| > 1, *p*-value < 0.05, *n* = 6).

In this within-subject study, principal component analysis (PCA) and orthogonal partial least squares discriminant analysis (OPLS-DA) were employed to assess metabolic differences between the WLO_Pre and WLO_Post groups. PCA revealed no distinct separation between groups, with complete overlap along the first principal component (Figure 6A). OPLS-DA demonstrated clear discrimination between WLO_Pre and WLO_Post samples (Figure 6B). Volcano plots were further used to identify serum metabolites significantly altered by WLO supplementation. A total of 1208 metabolites were identified from untargeted metabolomics platforms. The criteria used were |log_2_(FC)| > 1 and *p*-value < 0.05. The volcano plot revealed 8 significantly differential metabolites, of which 4 were upregulated and 4 were downregulated (Figure 6C). Detailed metabolite information is presented in Table S8, which summarizes the significantly altered serum metabolites—spanning neuropeptides, lipid derivatives (fatty acyls and alcohols), xenobiotics, and heme-related compounds—and suggests perturbations in neuroendocrine signaling, lipid metabolism, and heme metabolism.

#### 3.3.4. Fecal Microbiota

**Figure 7:**
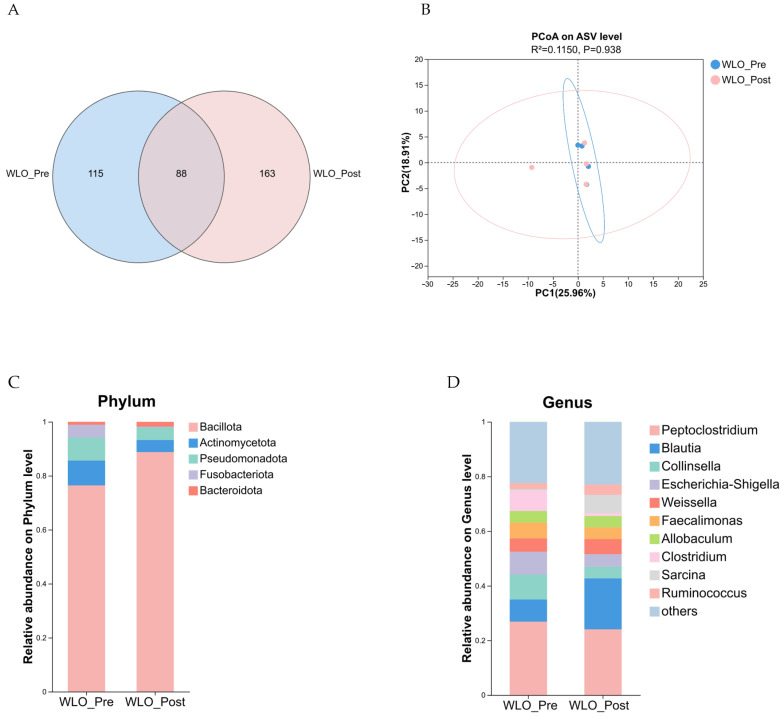
16S rRNA gene sequencing of fecal samples from the WLO_Pre and WLO_Post groups (*n* = 4). (**A**) Venn diagram illustrating the differentially abundant ASVs. (**B**) Principal coordinates analysis (PCoA) of beta diversity. (**C**) Taxonomic composition of fecal microbiota at the phylum level. (**D**) Taxonomic composition of fecal microbiota at the genus level.

At the ASV level, species richness in fecal samples from the WLO_Pre and WLO_Post groups of Shiba Inu was analyzed to determine the number of shared and unique species between the groups. Overall, the WLO_Pre group yielded 203 ASVs, while the WLO_Post group yielded 251 ASVs, with 88 ASVs shared between both groups (Figure 7A).

Alpha diversity metrics, including ACE, Chao, Shannon, Simpson, and Pielou’s evenness indices, revealed no significant differences among groups (Table 11; *p* < 0.05). Principal coordinate analysis (PCoA) based on Binary Euclidean distance, combined with permutational multivariate analysis of variance (ADONIS), showed no significant separation between the WLO_Pre and WLO_Post groups (Figure 7B; R^2^ = 0.1150, *p* = 0.938), indicating that WLO supplementation did not significantly affect the overall fecal microbiota structure in Shiba Inu dogs.

The five most abundant phyla collectively accounted for over 99% of the microbiota, including Bacillota, Actinomycetota, Pseudomonadota, Fusobacteriota, and Bacteroidota (Figure 7C). In the WLO_Post group, the relative abundance of Bacillota significantly increased by 16.16% compared to the WLO_Pre group (Table 12; *p* < 0.05), whereas Actinomycetota decreased by 51.52%, Pseudomonadota by 44.31%, and Fusobacteriota by 95.14%. Bacteroidota exhibited a non-significant increase of 60.91%. To investigate microbiota alterations at the genus level, taxonomic annotation was performed at this resolution. The relative abundances at the genus level indicated that the top 8 abundant genera included *Peptoclostridium*, *Blautia*, *Collinsella*, *Escherichia-Shigella*, *Weissella*, *Faecalimonas*, *Allobaculum*, and *Clostridium* (Figure 7D). In the WLO_Post group, the relative abundance of *Blautia* significantly increased by 128.83% compared to the WLO_Pre group (Table 13; *p* < 0.05), whereas several genera exhibited non-significant changes: *Weissella* increased by 12.91%, whereas *Peptoclostridium* decreased by 10.39%, *Collinsella* by 53.25%, *Escherichia-Shigella* by 45.16%, *Faecalimonas* by 26.12%, *Allobaculum* by 0.95%, and *Clostridium* by 86.41%.

#### 3.3.5. Spearman Correlation Analysis Between Fecal Bacteria and Differential Metabolites

**Figure 8:**
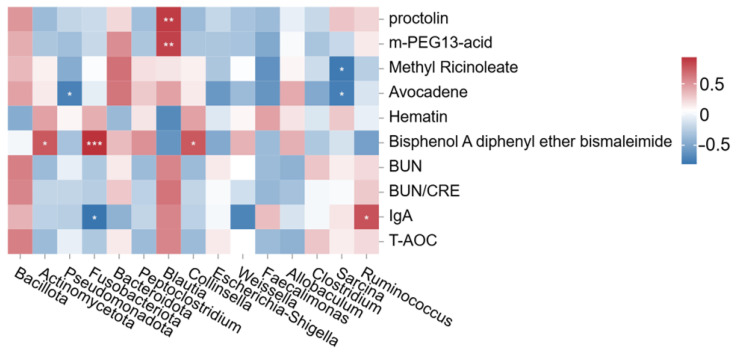
Spearman correlation heatmap between fecal bacteria and differential metabolites. The symbol (*) indicates a significant correlation between fecal bacteria and differential metabolites (* *p* < 0.05, ** *p* < 0.01, *** *p* ≤ 0.001). Red indicates a positive correlation, and blue indicates a negative correlation.

Correlation between fecal microbiota (at phylum and genus levels) and differential serum metabolites (including biochemical parameters) is shown in the heatmap (Figure 8). As shown, Actinomycetota exhibited a significant positive correlation with bisphenol A diphenyl ether bismaleimide, whereas Pseudomonadota showed a significant negative association with avocadene. Fusobacteriota displayed a significant positive correlation with bisphenol A diphenyl ether bismaleimide and a significant negative correlation with IgA. At the genus level, *Blautia* was significantly positively correlated with proctolin and m-PEG13-acid, *Collinsella* with bisphenol A diphenyl ether bismaleimide, and *Ruminococcus* with IgA, while *Sarcina* exhibited significant negative correlations with methyl ricinoleate and avocadene. These patterns suggest potential microbiota-driven modulation of xenobiotic, lipid, and immune-related pathways.

## 4. Discussion

The present study evaluated the short-term effects of whole lamb omasum (WLO) supplementation in healthy adult Shiba Inu dogs by integrating nutrient digestibility assessment, fecal characterization, serum biochemistry profiling, metabolomics, and 16S rRNA gene sequencing of fecal microbiota. The findings support the working hypothesis that WLO, as a fiber-rich raw ingredient for manufacturing dietary supplements in dogs, improves nutrient digestibility, enhances immune and antioxidant indices, and promotes beneficial shifts in the gut microbiota, without eliciting adverse physiological effects over the 10-day experimental period. These results are consistent with emerging evidence in canine nutrition, suggesting that diet composition can modulate metabolic and microbial homeostasis [12,30,31,32]. The observed responses highlight potential breed-specific adaptations in small breeds such as Shiba Inu, where metabolic rate and gastrointestinal physiology may differ from those of larger breeds [20,22,24,33]. Together, the between-group and within-subject analyses provide a comprehensive view of host–microbiota interactions, demonstrating rapid metabolic and microbial adaptations consistent with short-term dietary interventions in companion animals [34,35,36]. However, given the brief duration of supplementation, these beneficial effects are most likely transient. Future studies should incorporate larger sample sizes, multiple breeds, and extended durations to better assess the broader applicability and long-term benefits of WLO supplementation in canine nutrition.

Daily dry matter intake, gross energy intake, and body weight remained unaffected by WLO supplementation, indicating good compatibility with the basal diet and the absence of any impact on growth performance. This stability aligns with previous canine trials showing that short-term fiber supplementation maintains energy balance [31,37]. However, fecal scores decreased significantly, reflecting improved stool consistency and reduced moisture, likely due to the fibrous gastric matrix of WLO, which enhances water retention and modulates intestinal motility. Consistent with this, dietary fiber supplementation has been shown to improve fecal form and consistency in dogs [10,12,38]. Fritsch et al. reported complete clinical resolution of chronic diarrhea in 68% of dogs after fiber supplementation [38], while Liang et al. demonstrated that 1% soluble corn fiber achieved optimal fecal scores [10], and Lee et al. observed firmer stools with fiber–prebiotic blends [12]. In short-term feeding trials, fecal pH and dry matter typically remain unchanged, likely due to the brief intervention period, modest supplementation level, and buffering capacity of a balanced basal diet [39,40,41]. Collectively, WLO supplementation imposes no additional energetic burden and exerts no adverse influence on body weight or overall energy balance.

WLO supplementation significantly enhanced the apparent digestibility of crude fat and crude fiber, with no observable effects on dry matter, crude protein, calcium, or phosphorus digestibility. This improvement may be attributed to fibrous components in WLO that facilitate lipid emulsification and fermentation, given that the ruminant stomach contains partially digested plant material rich in cellulolytic bacteria [42,43,44]. Similar enhancements in nutrient utilization have been reported in dogs receiving dietary fiber, where apparent total tract digestibility of macronutrients increased [31,45]. Nogueira et al. demonstrated that fiber and prebiotic inclusion improved total dietary fiber digestibility [31], while Silvio et al. found that higher levels of fermentable fiber (pectin) increased total tract and large intestinal dry matter digestibility but reduced crude protein digestibility [45]. The 30 g daily WLO dose used in this study produced comparable effects, though potential breed-specific differences in Shiba Inu should be considered. Nonetheless, variations in fiber source, inclusion rate, and breed-related digestive physiology may explain reports of reduced nutrient digestibility in some fiber-supplemented diets [12,32]. Collectively, these results highlight WLO as a functional fiber source that improves fat and fiber utilization while maintaining balanced overall nutrient digestibility in dogs.

Between-group and within-subject analyses revealed elevations in BUN (within normal limits), BUN/creatinine ratio, IgA, and T-AOC, indicating enhanced nitrogen metabolism, mucosal immunity, and antioxidant capacity, while hepatic and renal enzymes, serum proteins, and lipid parameters remained stable. The rise in BUN likely reflects increased protein catabolism from the organ meat components of WLO, consistent with high-protein diet studies showing similar changes without renal impairment [46,47,48]. Elevated IgA may indicate microbiota-driven immune activation, as short-chain fatty acids (SCFAs) are known to stimulate intestinal IgA synthesis through multiple immunomodulatory pathways [49,50,51,52]. Likewise, increased T-AOC aligns with evidence that dietary fiber supplementation enhances antioxidant capacity and attenuates inflammation [4,53]. However, interpretation of WLO’s immunomodulatory and antioxidative effects is limited by reliance on IgA as the sole immune marker and a restricted antioxidant profile (T-AOC, SOD, MDA), which may overlook cytokine signaling and other antioxidant enzymes such as GSH-Px and CAT. Despite these constraints, the findings suggest that short-term WLO supplementation supports immune and antioxidant functions, providing a rationale for future studies incorporating broader serological and molecular analyses. However, changes observed in short-term trials do not necessarily reflect long-term health outcomes; further long-term studies are required to substantiate the benefits of WLO.

Between-group analyses identified 13 differential metabolites (10 upregulated, 3 downregulated), including phytoestrogens, phospholipids, drug metabolites, and microbial compounds, suggesting potential alterations in endocrine, lipid, and energy metabolism. Within-subject analyses identified 8 differential metabolites (4 upregulated, 4 downregulated), including neuropeptides, lipid derivatives, xenobiotics, and heme compounds, suggesting potential alterations in neuroendocrine, lipid, detoxification, and heme metabolism. In the between-group study, upregulated phospholipids (PC(14:0/20:4), PE(20:2/22:5)) may enhance membrane fluidity and signaling, potentially alleviating canine inflammation. Recent metabolomics show elevated serum phospholipids correlate with improved lipid metabolism regulation, as in β-glucan-supplemented canine models where similar profiles boost resilience to oxidative stress [54,55]. Accumulation of oxidative metabolites like 9,10,13-TriHOME (trihydroxylated linoleic acid derivative) indicates increased lipid oxidation. Although these metabolites confer antimicrobial benefits via eicosanoid signaling, chronic upregulation may exacerbate oxidative stress, contributing to canine hepatopathies and enteropathies [56,57]. Upregulated phytoestrogens mimic estrogen and may prevent cancer and age-related disorders via endocrine modulation; however, they risk reproductive disruptions, including fertility issues, in exposed dogs [58,59]. Reducing fructoselysine levels, a key Maillard reaction product, may limit AGE buildup, easing oxidative stress and boosting metabolic function in dogs [60]. Downregulation of peptide fragments (WYRSPSSYYENL) and glycosides (O-methylaltropyranosyl-lanostadienyl talopyranoside) could attenuate pro-inflammatory pathways; however, their contributions to canine pathophysiology remain unexplored, requiring mechanistic validation [61]. In the within-subject study, upregulated neuropeptides (proctolin) may regulate gut motility and appetite via neuroendocrine modulation, promoting satiety without weight changes; this aligns with dietary peptides enhancing anorexigenic signaling in high-fat-fed dogs [62,63]. Upregulation of lipid derivatives like methyl ricinoleate—a ricinoleic acid methyl ester—facilitates β-oxidation, enhancing energy utilization and attenuating dyslipidemia; paralleling nutraceutical effects in hyperlipidemic models [64]. Downregulation of xenobiotics (bisphenol A diphenyl ether bismaleimide) and heme metabolites underscores enhanced detoxification, curtailing endocrine disruptors and protecting against reproductive toxicities in phytoestrogen contexts [65,66]. Avocadene downregulation may have complex implications for canine oxidative stress and metabolism, with benefits and risks [67]. In the metabolomics analysis, only the negative-ion mode was employed for serum metabolite profiling, which may limit the detection of certain positively charged. This methodological constraint represents a study limitation, and future research should incorporate both ionization modes for a more comprehensive metabolome coverage. Collectively, the observed metabolomic shifts indicate that WLO supplementation can beneficially modulate lipid and endocrine metabolism and attenuate oxidative stress, thereby contributing to improved systemic metabolic homeostasis in dogs. However, these metabolomic findings should be interpreted cautiously as hypothesis-generating rather than definitive, given the short-term nature of the intervention; the observed effects are likely transient and do not necessarily reflect sustained long-term health benefits.

Microbiota modifications can occur rapidly, with intervention type and host factors significantly influencing microbial composition, particularly in small breeds like Shiba Inu dogs [24]. WLO supplementation significantly increased Bacillota, favoring SCFA-producers vital for canine colonic health and energy metabolism [17]. This enrichment, without altering alpha or beta diversity, suggests selective effects, potentially boosting mucosal immunity via SCFAs like butyrate that enhance epithelial barriers [68]. Within-subject *Blautia* increases indicate probiotic benefits, reducing inflammation and improving mental health, as *Blautia* correlates with lower anxiety in dogs [69]. Non-significant rises in *Ruminococcus*, core microbiome members, may support fiber fermentation and SCFA production, aiding digestion in small breeds like Shiba Inu [70]. Decreases in Actinomycetota, Bacteroidota, and Fusobacteriota resemble BARF patterns, where raw foods favor *Fusobacterium* and *Clostridium* but reduce Bacteroidetes. However, WLO’s plant fibers likely tempered these shifts, similar to prebiotics maintaining stability [71,72]. These adaptations, with lasting post-supplementation changes and stable core ASVs, suggest enduring gut resilience [73]. Although beneficial for healthy adults, protein-fermenter overgrowth in raw-like diets poses long-term metabolic risks [74]. Positive Bacillota correlations with metabolites like PC(14:0/20:4), BUN, and IgA, plus negative Actinomycetota/Bacteroidota associations with immune markers, indicate microbiota-driven immune and metabolic improvements [75]. Genus associations (*Blautia* with proctolin, *Ruminococcus* with IgA) align with microbiome-metabolome interactions in IBD models. Similar heatmaps in reviews implicate SCFA-producers in anti-inflammatory pathways, highlighting WLO’s role in gut–brain–immune networks [76]. Collectively, these microbiota alterations indicate that WLO supplementation selectively enriches beneficial SCFA-producing taxa, thereby enhancing colonic barrier function, mucosal immunity, and systemic metabolic homeostasis while maintaining overall community stability.

Traditional commercial lamb green tripe products typically consist solely of cleaned tripe tissue, with the gastric contents removed during processing. Any health benefits of such products are therefore likely to arise primarily from nutrients intrinsic to the tripe tissue. By contrast, the WLO used in this study was prepared as slices comprising the entire sheep omasum together with its gastric contents, and thus contained a higher crude fiber content derived from retained plant material, which may have provided additional effects on gut function and the intestinal microbiota. A further practical implication is that WLO production omits the rinsing and emptying steps required for conventional tripe, potentially reducing processing costs and resource use. Nevertheless, the physiological responses observed in this trial should be interpreted as effects of this high-fiber WLO ingredient rather than of tripe tissue alone, and therefore may not be directly generalizable to Traditional commercial lamb green tripe products.

## 5. Conclusions

Short-term dietary supplementation with WLO in healthy adult Shiba Inu dogs improved apparent nutrient digestibility, fecal quality, immune status, antioxidant capacity, and induced favorable alterations in serum metabolome and fecal microbiota composition. Collectively, these multi-omics findings highlight WLO as a promising functional ingredient for canine diets, with particular potential to support gastrointestinal health, immune function, and metabolic homeostasis. However, long-term studies are required to confirm the sustained efficacy and broader applicability of WLO across different breeds and physiological states.

## Figures and Tables

**Table 1:** The nutrient composition of WLO.

Items	Content
Nutrient component ^1^	
Dry matter (%)	27
Crude protein (% DM)	39.63
Crude ash (% DM)	9.9
Crude fat (% DM)	11.5
Crude fiber (% DM)	17.1
Gross phosphorus (% DM)	1.03
Calcium (% DM)	1.09
Gross energy (Kcal/100 g)	133.3

^1^ All test methods were in accordance with the national standard.

**Table 2:** Effect of WLO on food (DM basis) and energy intake and body weight of Shiba Inu dogs between the CON_Post and WLO_Post groups (*n* = 6).

Parameter	CON_Post	WLO_Post	*p*-Value
Food intake, g DM/d	143.52 ± 3.82	145.15 ± 3.28	0.752
GE intake, kcal/d	705.67 ± 18.79	684.68 ± 16.12	0.416
Body weight, kg	10.77 ± 0.75	11.37 ± 0.71	0.570

**Table 3:** Effect of WLO on fecal characteristics of Shiba Inu dogs between the CON_Post and WLO_Post groups (*n* = 6).

Parameter	CON_Post	WLO_Post	*p*-Value
Fecal Scores	3.03 ± 0.09	2.76 ± 0.06	0.011
Fecal pH	6.29 ± 0.14	6.14 ± 0.13	0.451
Fecal Dry Matter (%)	37.57 ± 1.07	38.03 ± 1.26	0.786

**Table 4:** Effect of WLO on apparent nutrient digestibility of Shiba Inu dogs between the CON_Post and WLO_Post groups (*n* = 6).

Parameter	CON_Post	WLO_Post	*p*-Value
Dry matter	92.31 ± 0.29	91.86 ± 0.39	0.380
Crude protein	92.59 ± 0.40	93.50 ± 0.47	0.168
Crude fat	81.61 ± 1.11	84.63 ± 0.58	0.036
Crude fiber	68.20 ± 1.44	76.02 ± 1.92	0.009
Calcium	52.05 ± 2.66	51.39 ± 2.55	0.861
Phosphorus	88.02 ± 1.64	86.66 ± 1.64	0.573

**Table 5:** Effect of WLO on serum biochemistry of Shiba Inu dogs between the CON_Post and WLO_Post groups (*n* = 6).

Parameter	CON_Post	WLO_Post	*p*-Value
TP, g/L	68.88 ± 1.42	71.28 ± 1.57	0.461
ALB, g/L	36.90 ± 0.76	37.12 ± 0.48	0.814
GLB, g/L	31.98 ± 1.79	34.17 ± 1.90	0.422
TBIL, µmol/L	1.88 ± 0.32	1.63 ± 0.33	0.597
AST, U/L	25.50 ± 4.29	29.00 ± 3.63	0.547
ALT, U/L	48.17 ± 6.94	68.00 ± 12.08	0.180
AMY, U/L	806.33 ± 74.76	892.83 ± 85.87	0.465
CK, U/L	110.17 ± 7.58	112.83 ± 13.05	0.863
CREA, µmol/L	108.23 ± 5.66	107.80 ± 5.48	0.957
BUN, mmol/L	6.07 ± 0.32	8.62 ± 0.55	0.002
GLU, mmol/L	5.11 ± 0.33	4.58 ± 0.19	0.198
TG, mmol/L	0.85 ± 0.05	1.01 ± 0.06	0.121
Ca, mmol/L	2.51 ± 0.06	2.46 ± 0.02	0.374
PHOS, mmol/L	1.28 ± 0.11	1.34 ± 0.05	0.627
ALB/GLB	1.18 ± 0.07	1.10 ± 0.07	0.483
BUN/CRE	57.24 ± 4.97	80.30 ± 4.70	0.007
Ca × PHOS	3.22 ± 0.24	3.30 ± 0.14	0.767
AST/ALT	0.54 ± 0.06	0.48 ± 0.09	0.596
IgA, ng/mL	14.22 ± 1.67	19.35 ± 1.04	0.026
T-AOC, µmol/mL	1.31 ± 0.07	1.71 ± 0.15	0.040
SOD, U/mL	2.55 ± 0.17	2.95 ± 0.25	0.213
MDA, nmol/mL	19.56 ± 0.98	17.03 ± 1.43	0.174

**Table 6:** Effect of WLO on alpha diversity indices of fecal microbial communities of Shiba Inu dogs between the CON_Post and WLO_Post groups (*n* = 4).

Parameter	CON_Post	WLO_Post	*p*-Value
Ace	110.80 ± 34.98	80.50 ± 18.77	0.475
Chao	110.80 ± 34.98	80.50 ± 18.77	0.475
Shannon	2.99 ± 0.33	2.62 ± 0.32	0.459
Simpson	0.11 ± 0.02	0.16 ± 0.06	0.502
Pielou_e	0.65 ± 0.02	0.60 ± 0.05	0.435

**Table 7:** Effect of WLO on composition of fecal microbiota at the phylum level of Shiba Inu dogs between the CON_Post and WLO_Post groups (*n* = 4).

Parameter	CON_Post	WLO_Post	*p*-Value
Bacillota	74.65 ± 4.38	88.78 ± 1.89	0.025
Actinomycetota	10.22 ± 3.20	4.46 ± 1.89	0.172
Bacteroidota	8.18 ± 4.74	1.77 ± 1.18	0.271
Pseudomonadota	4.07 ± 3.40	4.75 ± 3.35	0.892
Fusobacteriota	2.72 ± 1.60	0.23 ± 0.15	0.217

**Table 8:** Effect of WLO on composition of fecal microbiota at the genus level of Shiba Inu dogs between the CON_Post and WLO_Post groups (*n* = 4).

Parameter	CON_Post	WLO_Post	*p*-Value
*Peptoclostridium*	21.90 ± 8.32	24.06 ± 12.84	0.892
*Blautia*	14.64 ± 3.99	18.65 ± 1.14	0.371
*Collinsella*	9.54 ± 3.21	4.25 ± 1.92	0.207
*Romboutsia*	9.35 ± 9.15	1.16 ± 0.88	0.407
*Enterococcus*	6.66 ± 6.56	3.60 ± 3.59	0.696
*Faecalimonas*	4.56 ± 3.55	4.30 ± 3.36	0.959
*Escherichia-Shigella*	3.24 ± 2.82	4.59 ± 3.35	0.767
*Bacteroides*	5.09 ± 3.00	1.74 ± 1.16	0.338

**Table 9:** Effect of WLO on food (DM basis) and energy intake and body weight of Shiba Inu dogs between the WLO_Pre and WLO_Post groups (*n* = 6).

Parameter	WLO_Pre	WLO_Post	*p*-Value
Food intake, g DM/d	140.86 ± 4.78	145.15 ± 3.28	0.432
GE intake, kcal/d	692.62 ± 23.48	684.68 ± 16.12	0.761
Body weight, kg	11.30 ± 0.73	11.37 ± 0.71	0.640

**Table 10:** Effect of WLO on serum biochemistry of Shiba Inu dogs between the WLO_Pre and WLO_Post groups (*n* = 6).

Parameter	WLO_Pre	WLO_Post	*p*-Value
TP, g/L	71.38 ± 1.32	71.28 ± 1.57	0.897
ALB, g/L	35.97 ± 0.79	37.12 ± 0.48	0.170
GLB, g/L	35.43 ± 1.59	34.17 ± 1.90	0.371
TBIL, µmol/L	1.57 ± 0.20	1.63 ± 0.33	0.872
AST, U/L	27.33 ± 2.87	29.00 ± 3.63	0.717
ALT, U/L	57.83 ± 11.92	68.00 ± 12.08	0.438
AMY, U/L	912.00 ± 50.87	892.83 ± 85.87	0.866
CK, U/L	113.50 ± 9.16	112.83 ± 13.05	0.963
CREA, µmol/L	111.38 ± 7.01	107.80 ± 5.48	0.520
BUN, mmol/L	6.15 ± 0.48	8.62 ± 0.55	0.004
GLU, mmol/L	4.72 ± 0.21	4.58 ± 0.19	0.512
TG, mmol/L	0.90 ± 0.06	1.01 ± 0.06	0.077
Ca, mmol/L	2.52 ± 0.02	2.46 ± 0.02	0.094
PHOS, mmol/L	1.39 ± 0.11	1.34 ± 0.05	0.709
ALB/GLB	1.03 ± 0.06	1.10 ± 0.07	0.222
BUN/CRE	57.05 ± 7.47	80.30 ± 4.70	0.002
Ca × PHOS	3.50 ± 0.28	3.30 ± 0.14	0.548
AST/ALT	0.55 ± 0.08	0.48 ± 0.09	0.408
IgA, ng/mL	14.25 ± 1.52	19.35 ± 1.04	0.016
T-AOC, µmol/mL	1.26 ± 0.05	1.71 ± 0.15	0.036
SOD, U/mL	2.59 ± 0.25	2.95 ± 0.25	0.227
MDA, nmol/mL	20.22 ± 1.74	17.03 ± 1.43	0.205

**Table 11:** Effect of WLO on alpha diversity indices of fecal microbial communities of Shiba Inu dogs between the WLO_Pre and WLO_Post groups (*n* = 4).

Parameter	WLO_Pre	WLO_Post	*p*-Value
Ace	66.50 ± 9.64	80.50 ± 18.77	0.532
Chao	66.50 ± 9.64	80.50 ± 18.77	0.532
Shannon	2.42 ± 0.16	2.62 ± 0.32	0.577
Simpson	0.17 ± 0.01	0.16 ± 0.06	0.925
Pielou_e	0.57 ± 0.02	0.60 ± 0.05	0.707

**Table 12:** Effect of WLO on composition of fecal microbiota at the phylum level of Shiba Inu dogs between the WLO_Pre and WLO_Post groups (*n* = 4).

Parameter	WLO_Pre	WLO_Post	*p*-Value
Bacillota	76.43 ± 4.07	88.78 ± 1.88	0.037
Actinomycetota	9.20 ± 3.79	4.46 ± 1.89	0.453
Pseudomonadota	8.53 ± 7.86	4.75 ± 3.34	0.729
Fusobacteriota	4.73 ± 4.16	0.23 ± 0.15	0.344
Bacteroidota	1.10 ± 0.70	1.77 ± 1.18	0.489

**Table 13:** Effect of WLO on composition of fecal microbiota at the genus level of Shiba Inu dogs between the WLO_Pre and WLO_Post groups (*n* = 4).

Parameter	WLO_Pre	WLO_Post	*p*-Value
*Peptoclostridium*	26.85 ± 8.96	24.06 ± 12.84	0.870
*Blautia*	8.15 ± 2.77	18.65 ± 1.14	0.039
*Collinsella*	9.09 ± 3.81	4.25 ± 1.92	0.447
*Escherichia-Shigella*	8.37 ± 7.89	4.59 ± 3.35	0.729
*Weissella*	4.88 ± 4.87	5.51 ± 5.51	0.945
*Faecalimonas*	5.82 ± 3.86	4.30 ± 3.36	0.800
*Allobaculum*	4.22 ± 2.51	4.18 ± 2.47	0.995
*Clostridium*	7.36 ± 6.07	1.00 ± 0.6	0.398

## Data Availability

The original contributions presented in this study are included in the article/Supplementary Materials. Further inquiries can be directed to the corresponding authors.

## Supplementary Materials

The following supporting information can be downloaded at: https://www.mdpi.com/article/10.3390/vetsci13010058/s1, Figure S1: WLO samples. (A) Vacuum-packaged WLO and (B) non-vacuum-packaged WLO. Table S1: The ingredient composition and nutrient levels of the basal diet. Table S2: Microbiological and pathogen safety profile of whole lamb omasum (WLO) treated with electron beam irradiation compared to untreated controls; Table S3: Quality control statistics of 16S rRNA sequencing data from fecal samples in Shiba Inu dogs; Table S4: Comparison of food (DM basis) and energy intake and body weight of Shiba Inu dogs between the CON_Pre and WLO_Pre groups (*n* = 6); Table S5: Comparison of serum biochemistry of Shiba Inu dogs between the CON_Pre and WLO_Pre groups (*n* = 6); Figure S2: PCoA based on microbiota beta diversity between the CON_Pre and WLO_Pre groups (*n* = 4); Table S6: Comparison of alpha diversity indices of Shiba Inu dogs between the CON_Pre and WLO_Pre groups (*n* = 4); Table S7: Differential serum metabolites between the CON_Post and WLO_Post groups (*n* = 6); Table S8: Differential serum metabolites between the WLO_Pre and WLO_Post groups (*n* = 6).
