# Supplementary material for: Effects of Dietary Supplementation with Whole Lamb Omasum on Gut Health and Metabolism in Shiba Inu Dogs
Source: Vet Sci. 2026 Jan 7;13(1):58. doi: 10.3390/vetsci13010058 (PMC12846557; doi:10.3390/vetsci13010058)
Supplement: Supplementary file 1 [file vetsci-13-00058-s001.zip › Table S8.pdf]

Table S8. Differential serum metabolites between the WLO_Pre and WLO_Post groups (n = 6).

ID	Classification	Name	Log_2FC	$-Log_{10}(p\text{-Value})$
1	Neuropeptides	Proctolin	2.8087	4.0506
2	Polyethers	M-PEG13-acid	4.1459	2.6231
3	Fatty acyls	Methyl Ricinoleate	4.3569	1.4768
4	Fatty alcohols	Avocadene	1.4402	1.5413
5	Zwitterionic sulfonic acids	Mes-Hyd-Sul	-1.5164	1.5027
6	Heme derivatives	Hematin	-1.6453	2.2388
7	Bismaleimide resin monomers	Bisphenol A diphenyl ether bismaleimide	-2.7363	1.4003
8	Organometallics	Ir(ppy)3	-2.3334	1.7575
