# Supplementary material for: Effects of Dietary Supplementation with Whole Lamb Omasum on Gut Health and Metabolism in Shiba Inu Dogs
Source: Vet Sci. 2026 Jan 7;13(1):58. doi: 10.3390/vetsci13010058 (PMC12846557; doi:10.3390/vetsci13010058)
Supplement: Supplementary file 1 [file vetsci-13-00058-s001.zip › Table S1.pdf]

Table S1. The ingredient composition and nutrient levels of the basal diet.

Items	Content
Ingredient (% as fed)	
Fresh chicken meat	65
Fresh duck meat	8
Sweet potato granules	5
Fresh chicken liver	5
Fresh chicken heart	4
Tomato powder	3
Dried apple	3
Psyllium husk	3
Tapioca starch	2
Dried carrot	0.5
Cellulose	0.5
Egg yolk powder	0.5
Fish oil	0.5
Total	100.00
Nutrient component [1]	
Dry matter (%)	93.70
Crude protein (% DM)	35.93
Crude ash (% DM)	7.70
Crude fat (% DM)	16.70
Crude fiber (% DM)	3.10
Gross phosphorus (% DM)	1.05
Calcium (% DM)	1.22
Gross energy (Kcal/100g)	491.70

[1] All test methods were in accordance with the national standard.
