# Supplementary material for: Effects of Dietary Supplementation with Whole Lamb Omasum on Gut Health and Metabolism in Shiba Inu Dogs
Source: Vet Sci. 2026 Jan 7;13(1):58. doi: 10.3390/vetsci13010058 (PMC12846557; doi:10.3390/vetsci13010058)
Supplement: Supplementary file 1 [file vetsci-13-00058-s001.zip › Table S2.pdf]

Table S2. Microbiological and pathogen safety profile of whole lamb omasum (WLO) treated with electron beam irradiation compared to untreated controls.

Items	Irradiated (3.0 kGy)	Non-Irradiated
Salmonella (/25g)	ND [1]	ND
Shigella (/25g)	ND	ND
Staphylococcus aureus (CFU/g)	<10	<10
Escherichia coli (CFU/g)	<10	2.1×10^{2}
Aerobic plate count (CFU/g)	<10	1.2×10^{4}
Molds count (CFU/g)	<10	70
Brucellosis	NO Ct [2]	-
Pseudorabies virus	NO Ct	-

[1] ND =Not detected.

[2] NO Ct = No cycle threshold (undetectable by qPCR).

Microbiological analyses were conducted in accordance with the following Chinese national standards (GB/SN/T): Salmonella (GB/T 13091-2018; Determination of Salmonella in feeds. National Standardization Administration of the People's Republic of China: Beijing, China, 2018.), Shigella (GB/T 8381.2-2005; Determination of Shigella in feeds. National Standardiza-tion Administration of the People's Republic of China: Beijing, China, 2005.), Staphylo-coccus aureus (GB 4789.10-2016; National food safety standard—Food microbiological examination: Staphylococcus aureus. National Health and Family Planning Commission of the People's Republic of China: Beijing, China, 2016.), Escherichia coli (GB 4789.38-2012; National Food Safety Standard—Food Microbiological Examination: Enumeration of Escherichia coli. Ministry of Health of the People's Republic of China: Beijing, China, 2012.), aAerobic plate count (GB 4789.2-2022; National food safety standard—Microbiological examination of food: aerobic plate count. National Health Commission of the People's Republic of China: Beijing, China, 2022.), mMolds count (GB/T 13092-2006; Enumeration of molds count in feeds. National Standardization Administration of the People's Republic of China: Beijing, China, 2006.), Brucellosis (SN/T 4463-2016; Test of Brucellosis with real-time PCR at frontier port. General Ad-ministration of Quality Supervision, Inspection and Quarantine of the People's Repub-lic of China: Beijing, China, 2016.), and Pseudorabies virus (GB/T 35911-2018; Re-al-time PCR method for detection of pseudorabies virus. National Standardization Administration of the People's Republic of China: Beijing, China, 2018.).
