# Supplementary material for: Effects of Dietary Supplementation with Whole Lamb Omasum on Gut Health and Metabolism in Shiba Inu Dogs
Source: Vet Sci. 2026 Jan 7;13(1):58. doi: 10.3390/vetsci13010058 (PMC12846557; doi:10.3390/vetsci13010058)
Supplement: Supplementary file 1 [file vetsci-13-00058-s001.zip › Table S3.pdf]

Table S3. Quality control statistics of 16S rRNA sequencing data from fecal samples in Shiba Inu dogs.

Sample	Optimized Sequences	Denoised Sequences	Retention Rate (%)
CON_Pre1	61502	38567	62.71
CON_Pre2	61754	44892	72.69
CON_Pre3	64640	40210	62.21
CON_Pre4	57532	36309	63.11
CON_For1	64881	46957	72.37
CON_For2	61262	37953	61.95
CON_For3	60011	38045	63.40
CON_For4	58853	37695	64.05
LGT_Pre1	57041	42832	75.09
LGT_Pre2	60401	37899	62.75
LGT_Pre3	60719	44567	73.40
LGT_Pre4	56611	43827	77.42
LGT_For1	64240	47700	74.25
LGT_For2	62728	45435	72.43
LGT_For3	62735	39893	63.59
LGT_For4	61748	38735	62.73
