# Supplementary material for: Effects of Dietary Supplementation with Whole Lamb Omasum on Gut Health and Metabolism in Shiba Inu Dogs
Source: Vet Sci. 2026 Jan 7;13(1):58. doi: 10.3390/vetsci13010058 (PMC12846557; doi:10.3390/vetsci13010058)
Supplement: Supplementary file 1 [file vetsci-13-00058-s001.zip › Table S4.pdf]

Table S4. Comparison of food (DM basis) and energy intake and body weight of Shiba Inu dogs between the CON_Pre and WLO_Pre groups (n = 6).

Parameter	CON_Pre	WLO_Pre	p-Value
Food intake, g DM/d	133.37±3.73	140.86±4.78	0.244
GE intake, kcal/d	655.76±18.34	692.62±23.48	0.244
Body weight, kg	10.70±0.73	11.30±0.73	0.574
