# Supplementary material for: Effects of Dietary Supplementation with Whole Lamb Omasum on Gut Health and Metabolism in Shiba Inu Dogs
Source: Vet Sci. 2026 Jan 7;13(1):58. doi: 10.3390/vetsci13010058 (PMC12846557; doi:10.3390/vetsci13010058)
Supplement: Supplementary file 1 [file vetsci-13-00058-s001.zip › Table S5.pdf]

Table S5. Comparison of serum biochemistry of Shiba Inu dogs between the CON_Pre and WLO_Pre groups ($n = 6$).

Parameter	CON_Pre	WLO_Pre	*p*-Value
TP, g/L	70.62±1.48	71.38±1.32	0.707
ALB, g/L	36.38±0.97	35.97±0.79	0.746
GLB, g/L	34.23±1.62	35.43±1.59	0.609
TBIL, μmol/L	1.82±0.22	1.57±0.20	0.414
AST, U/L	23.50±5.03	27.33±2.87	0.523
ALT, U/L	43.83±5.02	57.83±11.92	0.325
AMY, U/L	740.50±71.99	912.00±50.87	0.080
CK, U/L	95.67±9.97	113.50±9.16	0.217
CREA, μmol/L	96.20±7.62	111.38±7.01	0.173
BUN, mmol/L	5.61±0.76	6.14±0.48	0.394
GLU, mmol/L	4.92±0.45	4.72±0.21	0.706
TG, mmol/L	1.05±0.23	0.90±0.06	0.555
Ca, mmol/L	2.43±0.05	2.52±0.02	0.139
PHOS, mmol/L	1.31±0.14	1.39±0.11	0.656
ALB/GLB	1.08±0.06	1.03±0.06	0.601
BUN/CRE	59.05±6.72	57.05±7.47	0.846
Ca×PHOS	3.15±0.29	3.50±0.28	0.406
AST/ALT	0.51±0.08	0.55±0.08	0.776
IgA, ng/ml	14.10±1.29	14.25±1.52	0.941
T-AOC, μmol/mL	1.30±0.10	1.26±0.05	0.715
SOD, U/ml	2.41±0.15	2.59±0.25	0.554
MDA, nmol/ml	19.18±1.36	20.22±1.74	0.646
