# Supplementary material for: Effects of Dietary Supplementation with Whole Lamb Omasum on Gut Health and Metabolism in Shiba Inu Dogs
Source: Vet Sci. 2026 Jan 7;13(1):58. doi: 10.3390/vetsci13010058 (PMC12846557; doi:10.3390/vetsci13010058)
Supplement: Supplementary file 1 [file vetsci-13-00058-s001.zip › Table S6.pdf]

Table S6. Comparison of alpha diversity indices of Shiba Inu dogs between the CON_Pre and WLO_Pre groups (n = 4).

Parameter	CON_Pre	WLO_Pre	*p*-Value
Ace	74.25±6.58	66.50±9.64	0.531
Chao	74.25±6.58	66.50±9.64	0.531
Shannon	2.50±0.03	2.42±0.16	0.686
Simpson	0.17±0.02	0.17±0.01	0.886
Pielou_e	0.58±0.02	0.58±0.02	0.847
