# Supplementary material for: Effects of Dietary Supplementation with Whole Lamb Omasum on Gut Health and Metabolism in Shiba Inu Dogs
Source: Vet Sci. 2026 Jan 7;13(1):58. doi: 10.3390/vetsci13010058 (PMC12846557; doi:10.3390/vetsci13010058)
Supplement: Supplementary file 1 [file vetsci-13-00058-s001.zip › Table S7.pdf]

Table S7. Differential serum metabolites between the CON_Post and WLO_Post groups (n = 6).

ID	Classification	Name	Log_2FC	$-Log_{10}(p\text{-Value})$
1	Phenylpropanoids	Coumesterol	1.5628	1.3082
2	Glycerophospholipids	PC(14:0/20:4)	1.0117	1.9110
3	Glycerophospholipids	PE(20:2/22:5)	1.0512	1.7119
4	pyrrolidone carboxylic acid	UCB-L 057	1.0142	1.4969
5	Fatty acyls	Icosanedioic acid	1.2007	1.3803
6	Fatty acyls	8,9,10-TriHOME	1.4733	1.6883
7	Carbohydrates	Fructoselysine	1.6077	2.1887
8	Cofactors	Dihydrolipoamide	1.3342	1.3125
9	Benzamide derivatives	Sildenafil-derived benzyl benzamide	1.112	2.0873
10	Terpenoids	Ganoleuconin N	1.1561	2.0780
11	Peptides	WYRSPSSYYENL	-1.1654	1.9818
12	Terpenoid glycosides	O-Methylaltropyranosyl-lanostadienyl talopyranoside	-1.2822	1.4244
13	Phosphoramidites	Bn-DMG-amidite	-1.4288	1.8761
