# Supplementary figures and images for: Effects of Dietary Supplementation with Whole Lamb Omasum on Gut Health and Metabolism in Shiba Inu Dogs
Source: Vet Sci. 2026 Jan 7;13(1):58. doi: 10.3390/vetsci13010058 (PMC12846557; doi:10.3390/vetsci13010058)

A

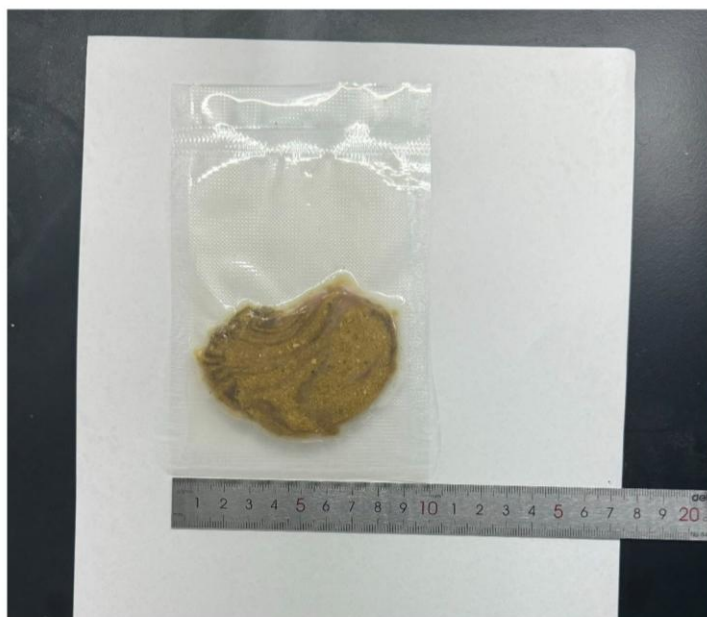

B

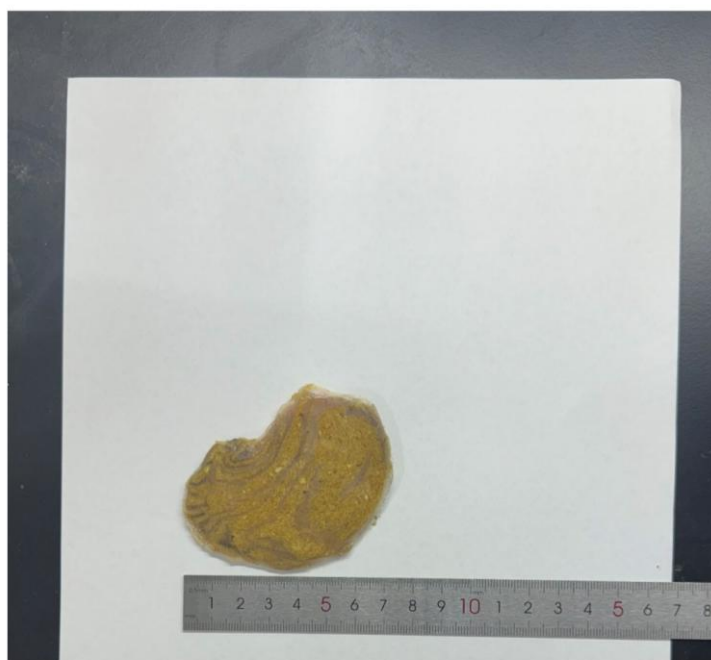

**Figure S1.** WLO samples. (A) vacuum-packaged WLO and (B) non-vacuum-packaged WLO.

Supplement: Supplementary file 1 [file vetsci-13-00058-s001.zip › Figure S1.pdf]

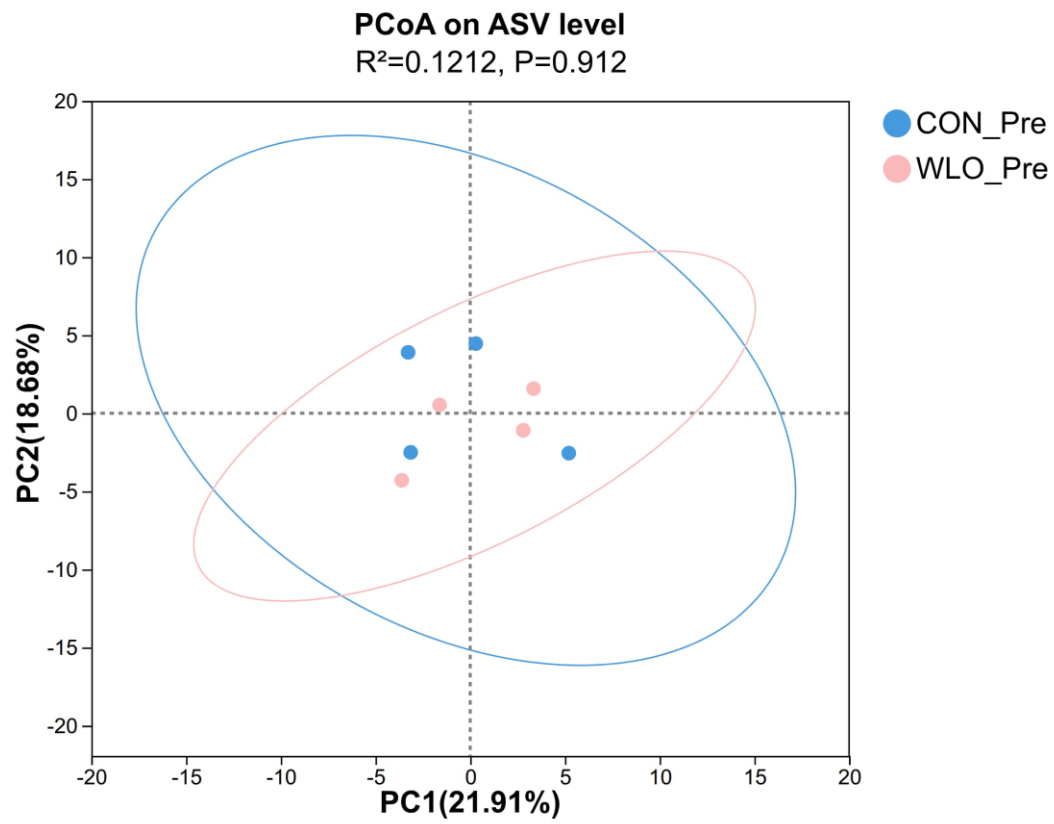

**Figure S2.** PCoA based on microbiota beta diversity between the CON\_Pre and WLO\_Pre groups ( $n = 4$ ).

Supplement: Supplementary file 1 [file vetsci-13-00058-s001.zip › Figure S2.pdf]
